# 3D Bioprinting-Based Vascularized Tissue Models Mimicking Tissue-Specific Architecture and Pathophysiology for *in vitro* Studies

**DOI:** 10.3389/fbioe.2021.685507

**Published:** 2021-05-31

**Authors:** Dong Gyu Hwang, Yoo-mi Choi, Jinah Jang

**Affiliations:** ^1^School of Interdisciplinary Bioscience and Bioengineering, Pohang University of Science and Technology, Pohang, South Korea; ^2^Department of Convergence IT Engineering, Pohang University of Science and Technology, Pohang, South Korea; ^3^Department of Mechanical Engineering, Pohang University of Science and Technology, Pohang, South Korea; ^4^Institute of Convergence Science, Yonsei University, Seoul, South Korea

**Keywords:** 3D bioprinting, 3D *in vitro* tissue model, vascularization, disease modeling, organ-organ crosstalk

## Abstract

A wide variety of experimental models including 2D cell cultures, model organisms, and 3D *in vitro* models have been developed to understand pathophysiological phenomena and assess the safety and efficacy of potential therapeutics. In this sense, 3D *in vitro* models are an intermediate between 2D cell cultures and animal models, as they adequately reproduce 3D microenvironments and human physiology while also being controllable and reproducible. Particularly, recent advances in 3D *in vitro* biomimicry models, which can produce complex cell structures, shapes, and arrangements, can more similarly reflect *in vivo* conditions than 2D cell culture. Based on this, 3D bioprinting technology, which enables to place the desired materials in the desired locations, has been introduced to fabricate tissue models with high structural similarity to the native tissues. Therefore, this review discusses the recent developments in this field and the key features of various types of 3D-bioprinted tissues, particularly those associated with blood vessels or highly vascularized organs, such as the heart, liver, and kidney. Moreover, this review also summarizes the current state of the three categories: (1) chemical substance treatment, (2) 3D bioprinting of lesions, and (3) recapitulation of tumor microenvironments (TME) of 3D bioprinting-based disease models according to their disease modeling approach. Finally, we propose the future directions of 3D bioprinting approaches for the creation of more advanced *in vitro* biomimetic 3D tissues, as well as the translation of 3D bioprinted tissue models to clinical applications.

## Introduction

The establishment of effective methods for disease treatment and prevention requires a clear understanding of pathophysiological phenomena. Various experimental models that mimic human physiology have been developed to test the safety and efficacy of potential therapeutics (Benam et al., 2015; Weinhart et al., 2019). Conventional 2D cell culture models are the most widely used tool in laboratories and have thus provided critical insights into various research fields by reproducing fundamental biological functions or features *in vitro*. This approach is not only easy to control but also renders robust results in a quick and cost-efficient manners (Langhans, 2018). Moreover, the advent of stem cell engineering has enabled the generation of various lineages of human cells with specific genetic characteristics (Hoes et al., 2019). Nonetheless, cells on a 2D surface do not behave in the same way as they naturally do in a 3D microenvironment (Amelian et al., 2017; Langhans, 2018). On the other hand, animal models can be used to characterize complex pathophysiological mechanisms *in vivo*. However, although these models have greatly contributed to our current understanding of various diseases and potential treatments, *in vivo* experiments are less reproducible due to inter-individual variations and are more cost- and time-consuming compared to cell culture experiments (Healy, 2018). Efforts have been made to bridge the gap between humans and animals using humanized mouse models, which are implanted with functional human cells and tissues; however, this approach still entails important limitations due to species-specific differences (e.g., residual innate immune system, cytokines, and humoral responses) (Walsh et al., 2017). Moreover, ethical concerns exist regarding the use of animal models (Walker and Eggel, 2020). Therefore, the pressing need for alternative platforms to investigate human pathophysiology *in vitro* has led to the development of 3D *in vitro* tissue models.

Because 3D models are based on cell culture, those are not only easy to control and reproduce experimental conditions but also provide a 3D microenvironment that mimics the physiological microenvironment, thereby allowing cell-cell and cell-matrix interactions akin to the physiological ones. More importantly, this approach enables the modeling of human physiology when human-derived cell sources are used. Therefore, 3D *in vitro* tissues exhibit more natural cellular behaviors, morphology, and functions compared to conventional 2D cell culture models by mimicking the native microenvironment and cell scaffolding structures (Kim J. et al., 2020). Multicellular spheroid structures (hereinafter referred to simply as “spheroids”) allow the modeling of heterogeneous cell-cell interactions by aggregating multiple types of cells (Langhans, 2018; Ide et al., 2020; Kim M. et al., 2020). Although spheroids can resemble the characteristics of native tissue in terms of cellular behaviors including proliferation, differentiation, maturation, and migration, simulating complex structures using these models can be quite challenging (Chatzinikolaidou, 2016; Hoes et al., 2019; Salaris and Rosa, 2019). 3D *in vitro* models constructed using microengineering such as micropattern and microfluidic channels enable the spatial organization of cells with micron-scale precision (Bhatia and Ingber, 2014; Laurent et al., 2017). This approach allows cells to self-organize by providing the geometric cues of the native tissue. However, more advanced fabrication methods are required to guide geometric cell morphology such as convoluted tubules, chamber-like structures, and lobule-like structures, which would further enhance the structural maturity and function of 3D bioprinted tissues by reproducing the characteristics of tissue-specific analogs (Jang et al., 2018; Kim M. et al., 2020; Yong et al., 2020). Given these requirements, 3D bioprinting has been considered a promising fabrication method capable of placing biomaterials and cell-laden biomaterials, bioink, in the desired locations. Many studies have reported that 3D bioprinted tissue improved tissue/organ function by mimicking complex native tissue architectures (Choi et al., 2019; Kim M. et al., 2020; Kim J. et al., 2020; Yong et al., 2020).

Vascularization plays a pivotal role in achieving sizeable and complex *in vitro* models, because the tissue models with a thickness larger than 400 μm require vasculature to ensure cell viability. In addition, recapitulation of blood vessel is important to improve the similarity of tissue models by enriching the microenvironment and is important for observing crosstalk between blood vessels and organs (Auger et al., 2013; Pellegata et al., 2018). In this respect, 3D bioprinting technique facilitates the generation of vascularized *in vitro* tissue models because endothelial cells (ECs) can be positioned to form lumen structure effortlessly.

This review covers the latest trends in the fabrication of *in vitro* 3D bioprinted tissue models, as well as strategies to reproduce natural tissues/organs. Particularly, this review focused on blood vessels and highly vascularized organs such as the heart, liver, and kidney, as these organs are inherently related to the vascular system due to their specific metabolic activities and functions. Moreover, we provide a brief introduction to the representative functions and features of each organ and describe their relationship with blood vessels. We will then discuss 3D bioprinting approaches for the fabrication of 3D *in vitro* tissue models and the advantages of 3D bioprinting techniques for the recreation of physiological features of native organs. Furthermore, this review will also summarize the current state of disease modeling methods based on 3D bioprinted *in vitro* tissue models. Specifically, modeling approaches will be divided into three categories to facilitate their discussion: (1) chemical substance treatment, (2) 3D bioprinting of lesions, and (3) recapitulation of tumor microenvironments (TME). Finally, we discuss the remaining challenges of 3D bioprinting and propose potential strategies to achieve more realistic human pathophysiological features *in vitro* and translate 3D bioprinted tissue models to clinical applications.

## Mimicking Physiological Characteristics Using 3D Bioprinted *in vitro* Models

### Perfusable and Multi-Layered Blood Vessel Models in Various Sizes and Shapes

Blood vessels are responsible for the transport of substances such as nutrients, oxygen, hormones, and drugs to the cells, as well as the elimination of the metabolized substances from the cells. Moreover, blood vessels connect the organs of our body and allow them to interact. Blood vessels are typically classified as arteries, veins, and capillaries, and each vessel has its own unique characteristics in wall thickness, elasticity, lumen diameter, etc. (Dewhirst and Secomb, 2017; Jarvis, 2018). Existing *in vitro* blood vessel models were developed focusing on the transport of substances, and their functions are typically evaluated in terms of the barrier function and perfusion of vascular ECs. Although microfluidic techniques have been utilized to fabricate blood vessels by perfusing ECs through hollow channels, rectangular channels are not well-suited to mimic natural vascular structures as they lack structural complexity (Wong et al., 2012; Abudupataer et al., 2020; Cao et al., 2020).

3D bioprinting technology has enabled the fabrication of more realistic blood vessel models. Using sacrificial biomaterial ink, cylindrical hollow tubules were generated with perfusable vascular channels and multi-scale vascular networks (Bertassoni et al., 2014; Kolesky et al., 2014, 2016; Lee et al., 2014a,b). Moreover, ECs have been directly printed onto multi-layered blood vessels of different diameters using a co-axial printing approach (Jia et al., 2016; Gao et al., 2017; Pi et al., 2018).

**FIGURE 1:**
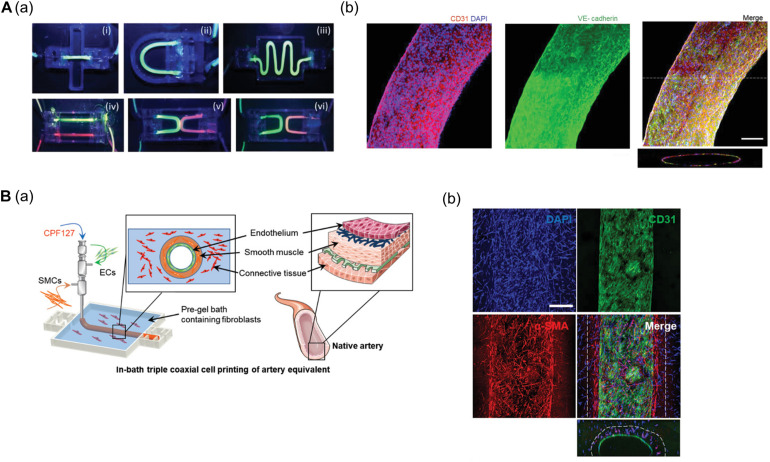
**(A)** Construction of perfusable and functional *in vitro* vascular models using co-axial cell printing. **(a)** Fabrication of various patterns of *in vitro* vascular models is demonstrated by perfusion of fluorescent dyes. **(b)** Expression of the cell-cell junction marker VE-cadherin is confirmed on day 7 in the printed vessels, indicating maturation of the vessels. Reprinted with permission from Gao et al. (2018). **(B)** Formation of triple-layered vascular model via in-bath triple co-axial cell printing. **(a)** Schematic describes printing procedure to produce triple-layered vascular model. **(b)** Immunofluorescence images show multi-layered blood vessels with endothelial cells (CD31) on the inside and surrounded by smooth muscle cells (α-SMA). Reprinted with permission from Gao et al. (2020).

Gao et al. (2018) established a freestanding, perfusable, and functional *in vitro* vascular model using co-axial printing and blood vessel-derived ECM bioink. This approach allowed not only for the creation of straight structures but also complex endothelium patterns according to predesigned geometry (Figure 1A). Shear stress can also be induced by providing perfusion through the hollow channel, resulting in improved selective permeability, thrombogenic quiescence, and self-remodeling. The authors further induced angiogenic sprouting and endothelium dysfunction by treating the 3D printed tissues with proangiogenic growth factors and inflammatory cytokines, respectively, thus mimicking native blood vessel pathophysiology (Gao et al., 2018). This vascular model was further developed to create separate endothelial and muscular layers using tri-axial printing, which more closely mimics the native vessel layers (Gao et al., 2019). More recently, the authors employed 3D in-bath coaxial cell printing to construct a triple-layered vascular model composed of connective tissue, smooth muscle, and endothelium, thereby enhancing barrier function (Figure 1B; Gao et al., 2020).

Extrusion-based bioprinting produces anatomically relevant vascular structures; however laser-based printing technologies [e.g., selective laser sintering (SLS), stereolithography (SLA), digital light processing (DLP), and 2-photon polymerization (2PP)] are more suitable for the production of capillary-sized microvessels that require higher printing resolution (Moroni et al., 2018a,b; Miri et al., 2019; Mota et al., 2020). Therefore, several studies have employed LAB (laser-associated bioprinting) to fabricate capillary-sized microvascular networks (Arakawa et al., 2017; Miri et al., 2019). Furthermore, dynamic conditions such as the cyclic movements of alveoli and coronary arteries are also important features of the physiological environment of blood vessels, and efforts have been made to mimic these movements *in vitro* (Huh et al., 2010; Stucki et al., 2018; Nam et al., 2020). For instance, Grigoryan et al. (2019) created a vascularized alveolar model that mimics complex microvessel networks using LAB and reproduces blood flow and oxygen delivery according to cyclic alveolar movements. The authors perfused deoxygenated red blood cells (RBCs) through the vessel inlet and allowed them to flow to an adjacent channel supplemented with oxygen via cyclic alveolar movements. The color of the RBCs shifted from dark to bright red, which indicated that the cells successfully performed gas exchange (Grigoryan et al., 2019).

As described above, 3D bioprinting techniques have proven to be a crucial strategy to mimic multi-layered blood vessel models. Particularly, these techniques allow for the production of blood vessel models with great structural complexity, as well as the generation of capillary-sized microvessel networks under tissue-specific microenvironments. Vascular morphology may vary due to tissue-specific differences; therefore, the capacity of ECs to generate a barrier varies dramatically among organs involved in absorption and filtration. Moreover, organ-specific vascular systems differ not only in permeability but also in their ability to deliver nutrients to tissues. For example, in energy-intensive organs such as the heart, ECs control nutrient delivery by adjusting capillary density. For example, EC permeability in the blood-brain barrier is highly selective, whereas endothelial permeability is very high in liver sinusoidal ECs (Potente and Mäkinen, 2017). Therefore, the production of organ-specific blood vessels and structurally robust and physiologically enhanced blood vessels can be a promising tool to connect 3D bioprinted organs. This approach would allow for a deeper understanding of organ-organ crosstalk and other physiological conditions such as absorption, distribution, metabolism, excretion, and cancer metastasis.

### Chamber-Like Cardiac Tissue Models Exhibiting Volume-Pressure Relationships

The heart is inherently related to the body’s blood vessel network. This organ is responsible for blood circulation throughout the body, supplying nutrients and oxygen and removing waste products through arteries and veins. To circulate blood, the myocardial muscle bundles are uniquely oriented. The myocardium consists of the endocardium, mid-wall, and epicardium, and these three parts are aligned in different directions to maximize the contraction. Moreover, given that the heart walls are composed of thick muscle bundles, they need to be supplied with sufficient oxygen and nutrients through the coronary arteries (Loukas et al., 2009; Lee A. et al., 2019; Noor et al., 2019). Many *in vitro* engineered heart tissue (EHT) models that recapitulate the function and physiology of cardiac tissues have been developed. These models generally exhibit the contractility and electrophysiological properties of native cardiac tissues (Leonard et al., 2018; Zhao et al., 2019; Goldfracht et al., 2020). However, the ECs used for the vascularization of cardiac tissues should be configured to create more biomimetic vasculatures. Furthermore, the volumetric features that recapitulate cardiac chambers must also be reproduced to understand the heart function and diseases associated with blood pumping (e.g., ejection fraction) (Li et al., 2018; Macqueen et al., 2018; Lee A. et al., 2019; Kupfer et al., 2020).

3D printing contributed to the fabrication of EHTs with precisely located cardiomyocytes (CMs) and stromal cells [e.g., ECs (Zhang et al., 2016; Arai et al., 2018; Maiullari et al., 2018) and fibroblasts (Arai et al., 2018; Anil Kumar et al., 2019; Daly et al., 2021)], thus mimicking the structural features of cardiac muscles and vasculatures (Wang Z. et al., 2018; Das et al., 2019; Yu et al., 2019). Furthermore, many studies have successfully developed volumetric cardiac chambers via support bath printing (Lee A. et al., 2019; Noor et al., 2019; Kupfer et al., 2020).

**FIGURE 2:**
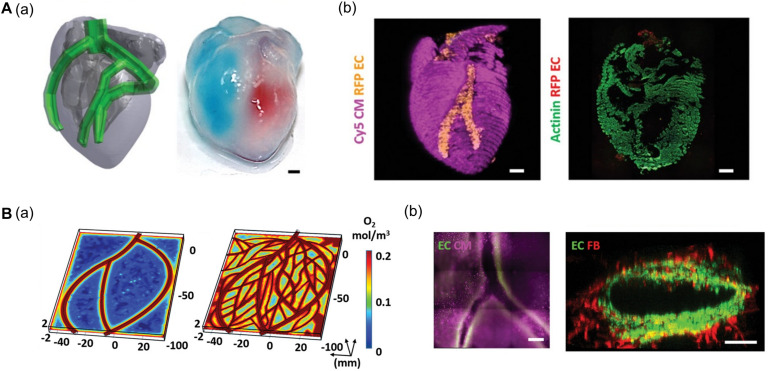
**(A)** Representative images of cardiac models composed of chamber and major vessel. **(a)** CAD design and printed structure of the cardiac chamber show the compartment of the left and right ventricle. **(b)** Confocal image of printed cardiac chamber indicates the spatial organization of CM (pink) and EC (orange) and cross-sectioned immunostaining showed internal compartmented CM (green) and EC (red) structure. **(B)** Characteristic of coronary artery network. **(a)** Supplemented blood vessels of coronary artery network exhibit improved oxygen diffusion via oxygen concentration profiles. **(b)** Immunofluorescence images show the branching and lumen structure of the coronary artery structure. Reprinted with permission from Noor et al. (2019).

Noor et al. (2019) generated a 3D cardiac chamber using a support bath strategy coupled with CT images to aid in the design of the printed structures (Figure 2A). The resulting cardiac chamber featured two separate chambers and a major blood vessel structure composed of induced pluripotent stem cell-derived cardiomyocytes (iPSC-CMs) and human umbilical vein endothelial cells (HUVECs), respectively. The separation of the chambers was demonstrated by filling them with different colored dyes. The authors also fabricated complex coronary artery networks using CAD data obtained from CT images. These experiments demonstrated that the resulting anatomically similar structure could supply oxygen to every area, taking into account the diffusion limits (Figure 2B; Noor et al., 2019). More recently, Kupfer et al. (2020) reported more advanced cardiac chamber constructs with two chambers and a vessel inlet and outlet with a high cell density. They supplemented photo-crosslinkable bioink (gelatin methacrylate and collagen methacrylate) with ECM components such as laminin-511/111 and fibronectin to regulate induced pluripotent stem cell (iPSC) behaviors (e.g., proliferation and differentiation). iPSCs were then embedded in modified bioink and directly printed to construct the cardiac chamber. The iPSCs then proliferated to a sufficient cell density and differentiated into CMs *in situ*. Remarkably, the cardiac chamber exhibited 3D features of native cardiac tissue including perfusion between chambers, volume-pressure relationships, and electromechanical functions, all of which are essential to the study of cardiac pathophysiology (Kupfer et al., 2020).

Although 3D bioprinted cardiac models have been proven too closely reproduce the volumetric features of natural tissues, their capacity to produce a volume differential is insufficient to mimic the pumping motion of the heart. Therefore, strategies to reproduce blood ejection are required. In this context, increasing the contractile force of cardiac tissue can enhance the movement of the cardiac chamber. Moreover, improving the maturity of CMs and increasing the thickness of the cardiac tissue can enhance the contractility of the cardiac chamber models at the cellular and tissue levels, respectively. Moreover, mimicking the anisotropic orientation of the myocardium can elevate the contractile capacity of cardiac chambers. Therefore, reproducing these features would facilitate the study of the pathophysiology related to blood pump function and hemodynamics.

### Hepatic Lobule-Like Models as Functional Units

The liver is an important organ that is largely responsible for the metabolic functions of the body (e.g., molecular anabolism, catabolism, and conversion and regulation of the energy balance), as well as detoxification and bile production. Two major blood vessels supply blood to the liver: 1) the hepatic artery and 2) the hepatic portal vein (Corsini and Bortolini, 2013; Kim J. et al., 2020; Ma et al., 2020). The liver is comprised of hexagonal structural and functional units called hepatic lobules which consist of a portal triad, hepatocytes arranged along a network of capillaries, and hepatic veins. Hepatic functions are highly specialized in each of the three zones of the hepatic lobules (zone 1: periportal; zone 2: midzonal area; zone 3: perivenous) (Ahn et al., 2019). Moreover, each zone has a unique microenvironment due to an oxygenation differential, which is determined by the distance from the hepatic arteries.

Current 3D *in vitro* liver tissue models have been developed to reproduce the 3D cell-cell/cell-matrix interaction, blood flow, and basic anatomical features of the liver, including hepatic zonation (Ahn et al., 2019; Collins et al., 2019). However, these features have been recreated as simple features or linear structures rather than complex and hexagonal structures. Therefore, additional studies are required to more properly reproduce the structural features of hepatic lobules including their complex vascular networks, zonal regions, and hexagonal functional units (Sacchi et al., 2020).

**FIGURE 3:**
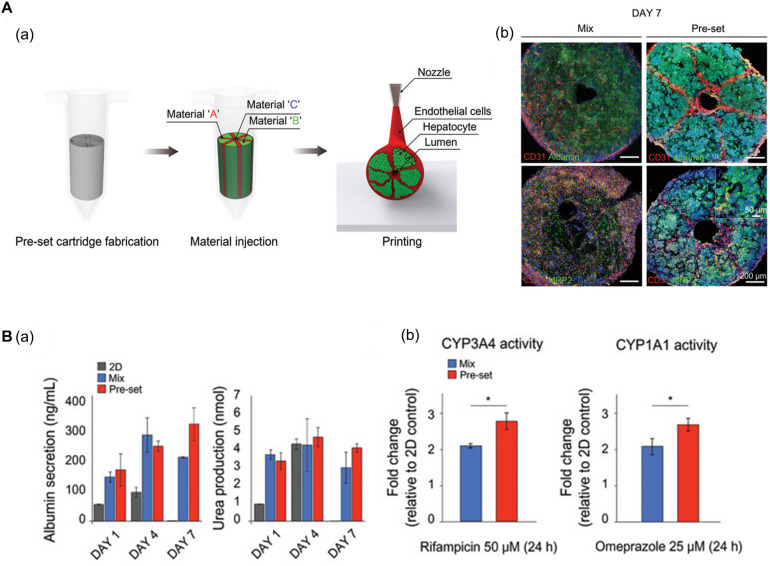
**(A)** Representative images of vascularized hepatic lobule structure. **(a)** The schematic of the preset extrusion bioprinting describes the printing strategy for hepatic lobules. **(b)** Immunofluorescence images of hepatic lobule construct using preset bioprinting exhibit well-preserved structural integrity not seen in the Mix group. **(B)** Evaluation of hepatic functions. **(a)** Albumin secretion and urea production measured by ELISA are enhanced in the printed hepatic lobule. **(b)** CYP3A4 and CYP1A1 enzyme activities are upregulated in printed lobule construct (*p < 0.05). Reprinted with permission from Kang et al. (2020).

Using 3D bioprinting techniques, several studies have successfully fabricated hexagonal and compartmentalized structures to mimic the anatomy of hepatic lobules (Ma et al., 2016; Grix et al., 2018; Yu et al., 2019; Kang et al., 2020; Mao et al., 2020). Kang et al. (2020) generated a vascularized hepatic lobule structure using preset extrusion bioprinting (Figure 3A). The resulting hepatic tissue contained hepatocytes surrounded and compartmentalized by ECs with a lumen structure mimicking the vascular network of a native structure. Unlike simple 3D mixtures of hepatic and ECs, the printed lobule exhibited higher liver function (e.g., albumin secretion and urea production), albumin, MRP2, and CD31 expression, and CYP3A4 and CYP1A1 enzyme activities. Furthermore, they used this printed lobule as a structural unit to produce larger hepatic lobule arrays using layer-by-layer printing (Figure 3B).

Furthermore, Mao et al. (2020) employed a digital light processing (DLP)-based 3D bioprinting approach to generate liver microtissues using tissue-specific bioink. The hydrogel of decellularized ECM (dECM) from porcine liver was mixed with gelatin methacrylate to obtain a tissue-specific microenvironment. Moreover, the microtissues were designed to recapitulate the microenvironment for the cellular function of the internal hepatocytes, which are derived from human fibroblasts, by increasing the contact area to mimic the environment of a capillary network. The authors concluded that the use of tissue-specific bioink and DLP-based 3D bioprinting resulted in a corresponding reproduction of the microenvironment and structural features of native liver tissue, thus enhancing cell viability and liver functions such as albumin and urea secretion (Mao et al., 2020).

The structural and functional features of liver lobules and vascular networks have been well developed using 3D bioprinting technology. However, mimicking the heterogeneous functions of hepatocytes according to zonal location and the intricate network of blood vessels that irrigate the liver (e.g., hepatic arteries and hepatic portal veins) are still challenging. Differentiation of hepatocytes with functional heterogeneity could be achieved via an oxygen gradient (van Wenum et al., 2018; Tonon et al., 2019). Therefore, 3D bioprinting strategies to create hepatic lobule models should incorporate oxygen gradients. Specifically, the generation of perfusable vascular channels, which deliver oxygenated blood to hepatocytes, in anatomically accordant positions would enable the differentiation of hepatocytes into specialized cells. Furthermore, simulating other liver functions such as bile production would result in more realistic *in vitro* liver models.

### Convoluted Renal Proximal Tubule Models With Vascular Interfaces

The kidney plays a major role in the filtration of various molecules and homeostasis regulation (Gupta et al., 2020). Nephrons, the functional units of the kidney, consist of Bowman’s capsule glomerulus, and tubules, such as the proximal and distal convoluted tubules and loop of Henle. Nephrons closely interact with blood vessels, such as the renal artery and vein (Wragg et al., 2019; Gupta et al., 2020). Given that kidney models are typically based on fluidic systems, several techniques are employed to manufacture 3D *in vitro* kidney models, including soft lithography, molding, and hollow fibers (Wilmer et al., 2016; Wragg et al., 2019; Zanetti, 2019; Singh et al., 2020). Although these models exhibited microfluidic conditions, they cannot fully mimic the complex tubule structure that may affect renal tubule cell performance *in vitro* (Wilmer et al., 2016; Wragg et al., 2019; Singh et al., 2020). 3D bioprinting research has largely focused on the fabrication of perfusable systems with convoluted structures to recapitulate the architecture and functions of native proximal tubules (Homan et al., 2016; Lin et al., 2019; Singh et al., 2020). Moreover, multiple renal cells could be elaborately positioned to mimic the complex cellular composition and functions of the native kidney tissues.

**FIGURE 4:**
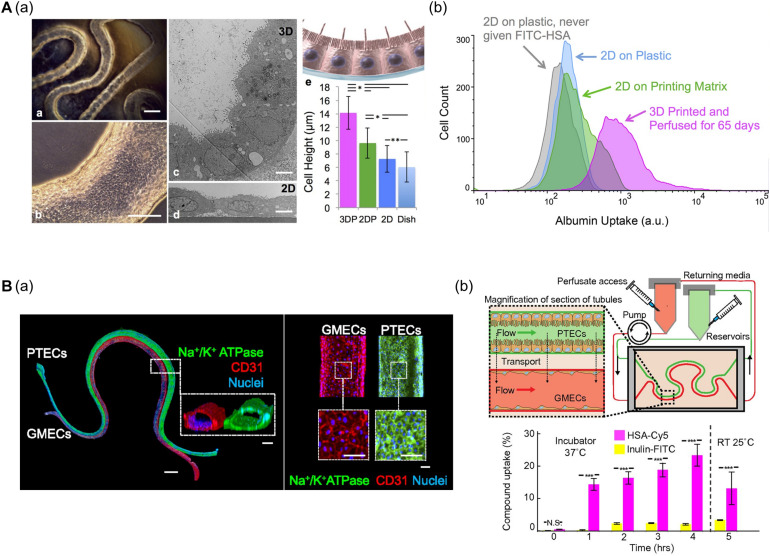
**(A)** Characteristics of 3D bioprinted convoluted renal proximal tubule structure. **(a)** Phase contrast and TEM images of printed proximal tubule models and enhanced cell height in 3D proximal tubule model. **(b)** Flow cytometry data demonstrated increased albumin uptake compared to 2D conditions (*p < 0.001; **p < 0.02). Reprinted with permission from Homan et al. (2016). **(B)** 3D renal proximal tubule with glomerular microvascular channels. **(a)** Whole-mount immunofluorescence images show the co-localized epithelium and endothelium with a distance of approximately 70 μm. **(b)** Schematic depicts a closed-loop perfusion system for assessment of renal resorption, and time-dependent measurements of albumin and insulin uptake performed by 3D printed proximal tubules indicate selective transport of albumin from PTEC to GMEC channels (N.S.: not significant; ***p < 0.0001). Reprinted with permission from Lin et al. (2019).

Homan et al. (2016) developed a convoluted proximal tubule using sacrificial Pluronic F-127 (PF-127) bioink (Figure 4A). A suspension of human proximal tubule epithelial cells (PTECs) was perfused through a channel after the removal of PF-127 to form the lumen structure. The PTECs formed a polarized epithelium and biologically relevant morphology (e.g., enhanced brush borders and cell height) under continuous flow which mimicked physiological shear stresses. Unlike PTEC monolayer models, the developed proximal tubule exhibited an improved albumin uptake, which is important for body fluid homeostasis (Homan et al., 2016).

Lin et al. (2019) (i.e., the same research group) fabricated vascularized proximal tubule models that exhibited renal reabsorption of albumin and glucose via proximal tubule-glomerular microvascular exchange (Figure 4B). Using a similar strategy, proximal tubules and glomerular microvascular channels were created using channel-specific cells. The epithelium and endothelium were co-localized with a separation distance of approximately 70 μm. Active reabsorption was demonstrated by circulating specific solutes and drugs through each channel. Importantly, the proximal tubule selectively reabsorbed albumin when simultaneously perfused with albumin and inulin (Lin et al., 2019).

It has also been reported that the kidney cells are embedded into kidney tissue-derived dECM bioink to recapitulate a tissue-specific microenvironment, thereby improving the sensitivity of the 3D kidney models to drug-induced toxicity (Wilmer et al., 2016). Recently, Singh et al. (2020) introduced a co-axial nozzle-based direct cell printing strategy for proximal tubule fabrication using kidney dECM bioink. Based on this approach, renal PTEC- and HUVEC-laden bioinks were printed into hollow tubes. Moreover, this approach could potentially be used to fabricate a complex hollow tubule with both mono- and bi-layers in a single step. This strategy could be further utilized for the construction of complex renal tissues including both monolayered proximal tubules and bilayer glomerulus structures. Consistent with other studies, each hollow channel exhibited upregulation of tissue-specific gene expression compared to their 2D culture counterparts. Furthermore, both tubes displayed an adequate barrier function and albumin transport from the proximal tubules to the blood vessels (Singh et al., 2020).

The *in vitro* models of renal proximal tubules with perfusable and complex tubular constructs have been developed using 3D bioprinting technologies. These studies have mainly focused on reabsorption and transportation functions, both of which occur in proximal tubules and glomeruli. 3D bioprinting provides a promising means to construct a complete *in vitro* nephron model consisting not only of proximal tubules but also other structures such as Bowman’s capsule, the loop of Henle, and distal tubules. 3D bioprinting technology has been developed to utilize multiple bioinks, thus enabling the creation of models using bioinks corresponding to each part of the nephron. In addition, printing parameters can be adjusted to simulate various diameters and shapes (e.g., straight or convoluted) for each tubule. Furthermore, a 3D bioprinting approach employing a co-axial nozzle can generate double- or triple-layer tubules to create multilayer tubule structures. Taken together, generation of complete *in vitro* nephron models can provide important insights into the pathophysiological characteristics of native kidney tissues.

## Disease Models

As described above, 3D bioprinting enables the creation of structurally and physiologically comparable 3D *in vitro* tissue models. Based on the advantages of 3D bioprinted tissue models, many researchers have exposed chemicals (e.g., drugs and cytokines), construct lesions, and use patient-derived cells to investigate and test the pathophysiological properties of the disease (Table 1).

**TABLE 1:** Disease modeling methods applied to 3D bioprinted *in vitro* models.

**Disease modeling method**	**Disease**	**Modeling factors**	**References**
Treatment of chemical substances	Cardiotoxicity	Doxorubicin	Zhang et al., 2016
	Hepatotoxicity	Acetaminophen	Bhise et al., 2016
	Hepatotoxicity	Trovafloxacin and levofloxacin	Nguyen et al., 2016
	Liver fibrosis	Methotrexate, thioacetamide, TGF-β1	Norona et al., 2016
	Liver fibrosis	Methotrexate, TGF-β1	Norona et al., 2019
	Hepatotoxicity	Acetaminophen	Lee H. et al., 2019
	Hyperglycemia in renal proximal tubule	High glucose (400 mg/dL) level	Lin et al., 2019
	Asthma	IL-13	Park et al., 2019
	Cardiotoxicity	Verapamil	Kupfer et al., 2020
	Hepatotoxicity	Acetaminophen	Lee et al., 2020
	Hepatotoxicity	Acetaminophen, diclofenac, nifedipine, trovafloxacin, indomethacin, methotrexate	Ide et al., 2020
3D Bioprinting of lesions	Cardiac fibrosis	Deposition of fibrotic spheroid	Daly et al., 2021
	Atherosclerosis	Printing parameter and printing path	Gao et al., 2020
Recapitulation of TME	Breast cancer	Breast cancer in the central region surrounded with ADMSC	Wang Y. et al., 2018
	Glioblastoma	Vascularized multicellular tumor spheroids	Han et al., 2020
	Glioblastoma	Paracrine and juxtracrine crosstalk between GBM and macrophages	Heinrich et al., 2019
	Glioblastoma	Recapitulating for hypoxia in GBM and using patient-derived GBM	Yi et al., 2019
	Breast cancer	Blood and lymphatic vessel pair	Cao et al., 2019
	Lung cancer Melanoma	Physically and chemically reconstructed to reproduce metastatic model	Meng et al., 2019
	Breast cancer	Cancer cell core surrounded by several stromal cell types	Langer et al., 2019

### Chemically Induced Disease Modeling

Exposure to bioactive compounds such as drugs and cytokines to induce tissue injuries (e.g., cardiotoxicity, hepatotoxicity, and renal toxicity) or other diseases (e.g., fibrosis and inflammation) is the easiest and most widely used method for disease modeling. Disease modeling methods based on drug-induced toxicity can be used to ensure that the 3D *in vitro* models respond appropriately to drugs or other bioactive compounds, thus highlighting the potential of 3D bioprinted tissue as a promising drug testing platform. Therefore, compounds that are already known to induce organ dysfunction or anticancer drugs with severe side effects are often used (Bhise et al., 2016; Nguyen et al., 2016; Norona et al., 2016, 2019; Zhang et al., 2016; Lee H. et al., 2019; Lee A. et al., 2019; Lee et al., 2020; Ide et al., 2020; Kupfer et al., 2020). Similarly, inflammatory factors such as inflammatory cytokines and other chemical substances are used to stimulate tissue models and induce pathological features such as inflammation and fibrosis (Norona et al., 2016, 2019; Lin et al., 2019; Park et al., 2019).

**FIGURE 5:**
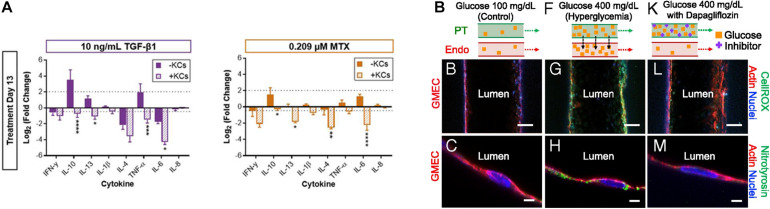
**(A)** Impact of Kupffer cells on the chemically induced inflammatory response of 3D bioprinted liver tissue models (*p < 0.05; **p < 0.01; ***p < 0.001; ****p < 0.0001). Reprinted with permission from Norona et al. (2019). **(B)** Pathophysiological features of GMEC lumen under normo- and hyperglycemic conditions indicated by immunofluorescen staining. Reprinted with permission from Lin et al. (2019).

Several recent studies have reported that 3D tissue models can be used to realistically mimic disease states. Norona et al. (2016) generated a 3D bioprinted liver tissue model composed of compartmentalized hepatocytes and stromal cells including stellate cells and ECs. This model was then treated with several fibrogenic compounds such as TGF-β1, methotrexate, and thioacetamide to induce liver fibrosis (Norona et al., 2016). The authors further enhanced the biomimetic properties of the liver tissue model by adding Kupffer cells, a type of inflammatory cell that mainly populates intact liver tissues and is responsible for homeostasis (Figure 5A). Subsequently, the author observed the response of the liver tissue model to the integrated Kupffer cells after inducing fibrogenesis as described in previous studies. The Kupffer cells in the liver-tissue model were observed to behave almost similarly as the Kupffer cells *in vivo*, behaving as immune cells under pathophysiological conditions. Concretely, the presence of Kupffer cells shortened the release of lactate dehydrogenase, increased collagen deposition, and altered cytokine responses (Norona et al., 2019).

As mentioned in the previous kidney tissue modeling section, Lin et al. (2019) established an *in vitro* proximal tubule model with a glomerular microvascular channel interface (Figure 5B). Furthermore, a reduction in glucose reabsorption was observed when Dapagliflozin [i.e., a type 2 diabetes medication known to inhibit glucose transporter (SGLT2) receptors] was administered, thus confirming that their model responded properly to the drug stimulus. They also induced their models to hyperglycemia by circulating a high glucose medium into the proximal tubule. Damage in both tubular channels was then observed, including a disruption of the cell-cell junction and lowered PTEC height. Interestingly, the vascular channel was restored when the tissue was treated with Dapagliflozin, which blocks glucose transport from the proximal tubule to the vasculature. These results indicated that the developed 3D printed tissues could serve as a platform for diabetes research.

### 3D Bioprinting of Disease Factors to Mimic Pathological Structures

*In vitro* disease models can be constructed by recreating the defining features of a specific disease using 3D bioprinting technology. Using this approach, spheroids can be deposited at precise locations as building blocks to create larger and more complex structures (Kizawa et al., 2017; Arai et al., 2018; Kim et al., 2018). Moreover, spheroids have also been utilized to model a variety of diseases such as fibrosis and tumor models with high cell densities. This approach has been used to generate oxygen gradients to recapitulate the disease microenvironment (Kim et al., 2018; Sacchi et al., 2020; Daly et al., 2021).

**FIGURE 6:**
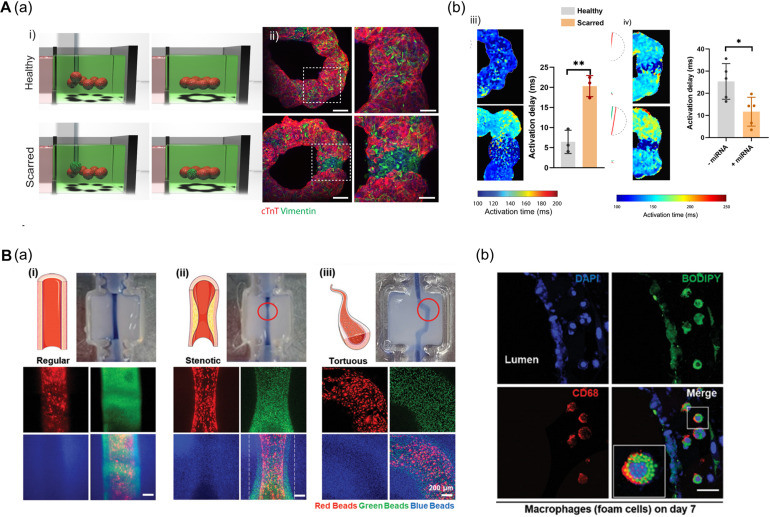
**(A)** 3D bioprinting of cardiac fibrosis models. **(a)** Schematic diagram of the procedure for creating a cardiac fibrosis model, and immunofluorescence image of cardiac fibrosis model. **(b)** Disrupted electrophysiological synchronization of cardiac models, and the therapeutic effect of miRNA treatment (*p < 0.05; **p < 0.01). Reprinted with permission from Daly et al. (2021). **(B)** Pathophysiological features of *in vitro* atherosclerosis model. **(a)** Structural features of the atherosclerosis model generated by adjusting the printing parameters and printing path are indicated by fluorescent beads. **(b)** Recapitulation of the early stages of atherosclerosis is verified by LDL accumulation and foam cell formation. Reprinted with permission from Gao et al. (2020).

In this context, Daly et al. (2021) fabricated a ring-shaped EHT by arranging cardiac spheroids in a self-healing hydrogel bath and inducing the fusion of spheroids (Figure 6A). To mimic healthy and fibrotic heart tissue, they generated spheroids with varying ratios of iPSC-CMs and cardiac fibroblasts (CFs) (4:1 iPSC-CMs to CFs ratio for healthy tissues and 1:4 for scarred tissue). The authors then deposited spheroids (8 healthy spheroids for normal tissue; 1 scarred spheroid among 7 healthy spheroids for the disease model) to fabricate a ring-shaped EHT. The cardiac fibrosis model showed reduced contraction amplitudes and disrupted electrophysiological synchronization of EHTs. Additionally, the developed EHTs were used to study microRNA (miRNA) therapeutics aimed at cardiac regeneration after myocardial infarction. The cardiac fibrosis models to screen a range of treatment durations and assess the effects of miRNA treatment. Interestingly, the miRNA treatment enhanced iPSC-CM proliferation, leading to improvement of contractility and electrophysiological integration of scarred heart tissue (Daly et al., 2021).

Another approach to recapitulate the pathophysiological features of 3D bioprinted tissues is to adjust the printing parameters including pneumatic pressure and the nozzle moving speed. Even if a structure was printed on the same path, the details of the structure may vary due to changes in the printing parameters. Gao et al. (2020) applied a triple-layered vascular model to fabricate an atherosclerosis disease model (Figure 6B). Based on a normal vascular model, the authors generated a stenotic structure by adjusting printing parameters, and a tortuous structure was created by adjusting the printing path. Turbulent flows were generated in the stenotic and tortuous models and it was confirmed that the turbulent flows induced endothelial dysfunction. Moreover, the authors recapitulated early atherosclerosis conditions such as LDL accumulation and foam cell formation by circulating LDL and monocytes through the vessel, indicating the potential of triple-layered vascular models as promising tools for drug testing (Gao et al., 2020).

### 3D Bioprinting Approaches for the Recapitulation of Tumor Microenvironments

Cancer is a very complex malignant tissue that is composed of heterogeneous cell populations and a unique genetic specificity in each patient. Moreover, mutations may occur during cancer progression, thus potentially modifying the tumor tissue structure or that of surrounding tissues (Hass et al., 2020). The tumor microenvironment (TME) is a representative feature of cancer that affects several cancer properties such as proliferation, invasion, and metastasis (Spill et al., 2016). The TME is composed of many factors, including ECM, blood vessels, and cancer-associated cells (e.g., fibroblasts, macrophages), which may vary depending on the tissue and anatomical location. Therefore, recapitulating tissue-specific TMEs plays an important role in developing *in vitro* cancer models. Specifically, peripheral blood vessels are a crucial part of the TME, which sprout from the surrounding vessels and supply nutrients to the cancer. Additionally, metastasis occurs through the blood vessels. Therefore, reconstruction of the blood vessels is critical when developing *in vitro* cancer models. *In vitro* cancer modeling has largely focused on investigating cancer cells that interact with the TME using transwell co-culture systems, 3D hydrogel cultures, or microfluidic devices (Chung et al., 2017; Nashimoto et al., 2017; Lee et al., 2018; Nguyen et al., 2019; Haase et al., 2020). However, although these methods have successfully recapitulated both metastasis and the juxtracrine and paracrine effect induced by surrounding cells, the structural and cellular complexity of the TME remains largely unexplored.

On the other hand, 3D bioprinting techniques have been demonstrated to improve the complexity of TME models by simultaneously printing different cell types, including cancer cells, fibroblasts, immune cells, ECs, and ECM biomaterials using multiple highly precise heads (Zhao et al., 2014; Wang Y. et al., 2018; Langer et al., 2019). Moreover, mimicking tissue-specific TME heterogeneity and tumorigenesis is an important consideration for *in vitro* cancer modeling. For example, KRAS-mutant pancreatic ductal adenocarcinoma (PDAC) activates the epidermal growth factor receptor (EGFR) by inducing an autocrine feed-loop and amplifies the signals required for PDAC development. However, EGFR deletion in colorectal cancer cells and non-small lung cancer cells does not prevent tumor formation. Therefore, signal transduction pathway regulation may vary depending on cancer types and tissue origins (Schneider et al., 2017). Recently, bioprinted *in vitro* cancer models have been demonstrated to closely simulate the properties of actual cancers, including their defining phenotypes and pathological characteristics and distinct tissue-specific TME heterogeneity (Yi et al., 2019; Kim E. et al., 2020; Yoon et al., 2020).

**FIGURE 7:**
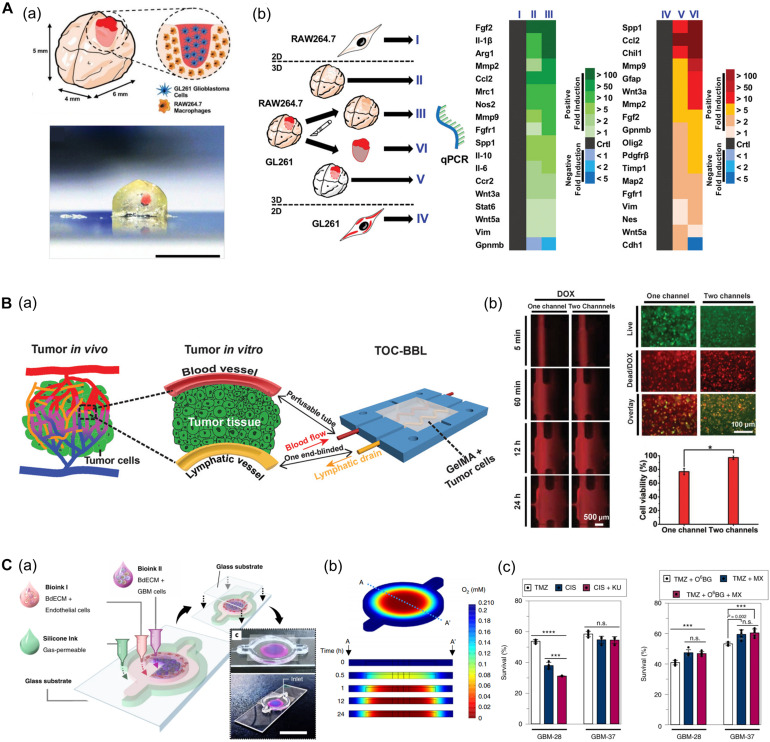
**(A)** GBM model based on a 3D bioprinted mini-brain construct containing macrophages. **(a)** Schematic and picture of GBM model exhibit GBM area in red on the bioprinted mini-brain. **(b)** Schematic illustrates the experimental conditions for the bioprinted RAW264.7/GL261 co-culture model and heatmap shows distinguished gene expression as a 2D monolayer (I and IV), 3D single bioprinted (II and V), and 3D bioprinted RAW264.7/GL261 co-culture model (III and VI) in RAW264.7 and GL261 glioblastoma. Reprinted with permission from Heinrich et al. (2019). **(B)** *In vitro* cancer model consisting of a pair of blood vessels and lymphatic vessels using 3D bioprinting. **(a)** Schematic shows the design of a bioprinted cancer model containing a pair of blood/lymphatic vessels that mimic actual TME. **(b)** Images on the left show the diffusion of DOX in a 1-channel/2-channel configuration, and the fluorescence images on the right exhibit cell viability for 24 h after DOX delivery (*p < 0.01). Reprinted with permission from Cao et al. (2019). **(C)** Application of patient-derived cell for *in vitro* GBM model mimicking hypoxic TME. **(a)** Schematic describes the design of GBM-on-a-chip with a compartmentalized cancer-vascular structure. **(b)** Colormap image of the oxygen gradient demonstrates the formation of central hypoxia in the GBM model. **(c)** Survival rate of patient-derived GBM treated with the drug combinations indicates patient-specific drug responses reflecting individual characteristics (n.s.: not significant; ***p < 0.001; ****p < 0.0001). Reprinted with permission from Yi et al. (2019).

Heinrich et al. (2019) developed a glioblastoma (GBM) model based on a mini-brain construct containing macrophages (Figure 7A). Specifically, a GBM area was printed on a section of the mini-brain construct. In this model, the authors observed the recruitment of macrophages in the GBM region and conversion to glioblastoma-associated macrophages, which was confirmed by the upregulation of typical phenotypes and related genes. Upon comparing the tissues from more than 150 patients with the 3D bioprinted tissues, it was confirmed that the behavior of macrophages in the proposed model was consistent with that of the patients, indicating its potential applicability in personalized medicine (Heinrich et al., 2019).

Furthermore, Meng et al. (2019) established an *in vitro* metastatic model via 3D bioprinting techniques, which were used to reproduce the structure of relevant peripheral blood vessels and growth factors (e.g., epidermal growth factor and vascular endothelial growth factor) released from stimuli−responsive capsules. A molecular gradient of growth factors was modulated using 3D bioprinting to mimic the biochemical characteristics of the TME. Lung cancer cells migrated to the blood vessels under the guidance of the growth factor gradient, and infiltrated the vascular system. As a result, the key features of cancer metastasis including invasion, intravasation, and angiogenesis were faithfully simulated. In addition, the usefulness of this cancer metastasis model for drug screening application was proved by assessing the anticancer efficacy of immunotoxins (Meng et al., 2019).

Among the factors that characterize the TME, lymphatic vessels are among the most closely related to cancer metastasis along with blood vessels. Moreover, microcirculation systems from blood vessels to lymphatic vessels provide a privileged recycling route to most anticancer drugs *in vivo*. In this regard, Cao et al. (2019) generated an *in vitro* cancer model consisting of a pair of blood vessels and lymphatic vessels using 3D bioprinting (Figure 7B). The pair of blood vessels and lymphatic vessels featured varying levels of diffusion properties of biomolecules and anticancer drugs in breast cancer cells. Given that anticancer drugs are delivered to the cancer cells through blood vessels and removed through the lymphatic vessels, their anticancer properties were attenuated with the incorporation of lymphatic vessels compared to when only blood vessels were included (Cao et al., 2019).

In particular, patient-derived cells retain physiological properties of *in vivo* lesions and represent the unique characteristics of patients. Therefore, these models have been utilized to investigate the patient-specific efficacy of and resistance to drugs and treatments. In this regard, recent studies using patient-derived cells are increasing. Yi et al. (2019) developed a bioprinted GBM model featuring a hypoxic environment, a representative feature of GBM. To achieve this, the authors printed patient-derived GBM cells that were encapsulated in brain dECM bioink concentrically on the cores, and then printed ECs around a core structure (Figure 7C). This GBM model showed a compartmentalized cancer-vascular structure that reproduced hypoxic conditions with a radial oxygen gradient. Remarkably, the results obtained with this bioprinted GBM model were consistent with the actual patient-specific resistance to chemoradiation and temozolomide (TMZ) combination therapy. Furthermore, the authors confirmed the potential utility of this model to screen for drug combinations associated with more efficient tumor resection (Yi et al., 2019).

Given that the development of treatment resistance in cancer is a process that takes several months, long-term culture of *in vitro* cancer models should be considered. Ozturk et al. (2019) developed a 3D vascular GBM model using sacrificial extrusion bioprinting. The researchers used 10% gelatin to generate two fluid vascular channels, after which HUVECs were seeded onto the channels. Subsequently, patient-derived GBM spheroids transduced with mCherry-expressing lentivirus were embedded between the channels. Thereafter, 100 μM of TMZ was perfused through the channels after 26 days of culture, and the authors observed tumor cell degeneration in the infiltrating area after 14 days. Vascularization enabled the tumor spheroids to grow for 26 days. Then, the spheroids had been treated with drugs for 37 days, allowing investigation of tumor behavior for up to 70 days. Interestingly, some GBM cells survived treatment and showed resistance to treatment, thus resuming proliferation and matrix invasion despite continued drug treatment. Therefore, the study demonstrated the feasibility of culturing the GBM model under vascularized TME conditions for up to 2 months, which offers important advantages for the study of long-term cancer cell behavior *in vitro*. Moreover, the authors demonstrated that cancer cell overgrowth is a mechanism of long-term TMZ treatment resistance (Ozturk et al., 2019).

3D bioprinting techniques have enhanced the complexity of the TME of *in vitro* cancer models. These 3D bioprinted *in vitro* cancer models have provided insights into the importance of recapitulating complex TME features in cancer research, as they allow for a more corresponding representation of patient-specific drug responsiveness and reduced drug efficacy according to TME conditions. Moreover, the combination of *in vitro* cancer models with multi-organ platforms can be applied to study cancer metastasis including extravasation and mesenchymal to endothelial transition, as well as endothelial to mesenchymal transition, invasion, intravasation, and angiogenesis.

## Conclusion and Future Outlook

3D bioprinting techniques have recently emerged as a promising tool to fabricate 3D *in vitro* tissue models through the precise arrangement of biomaterials and relevant cells based on their native tissue architecture (Choi et al., 2017; Kim J. et al., 2020). This review summarized the findings of recent studies related to 3D bioprinted tissue models that mimic blood vessel and various highly vascularized tissues, such as the heart, liver, and kidney. Although uncovered by this review, there are ongoing studies aiming to generate a 3D bioprinted intestine, lung, or skin model (Supplementary Table 1). Several studies have demonstrated the capacity of 3D bioprinting to recapitulate the geometric cues and complex cellular configurations of native tissues. Additionally, 3D bioprinting facilitates the research of tissue models, as it enables rapid prototyping, reproducibility, and repeatability. With this approach, many *in vitro* tissue models that recapitulate pathophysiological properties have been developed focused on basic functional or structural units of target tissues/organs.

Moving forward, several factors must be addressed to generate *in vitro* models with more biomimetic characteristics and to transform the *in vitro* system into clinical application. Therefore, to improve the quality of the printed constructs, future research must focus on the development of biomaterials with improved biofunctionality and printing fidelity, as well as fully functionalized cell sources that reflect personalized genotypes.

Scaling up the 3D bioprinted tissue models is a feasible goal in the near future. This could be achieved via modular tissue engineering, where small tissue modules (i.e., well-established 3D bioprinted functional units) are assembled to build large-scale engineered tissue models. Additionally, future models should incorporate more realistic vascularization in order to supply oxygen and nutrients to deeper cell layers beyond the diffusion limits.

Along with scale-up, the complete organ should in accomplished regarding cell composition as well as the anatomical structure. For now, among the various cell types, the application of organoids with cellular diversity could potentially improve cell composition restrictions. For instance, Kupfer et al. (2020) bioprinted iPSCs directly into a cardiac chamber structure and differentiated them into CMs. They observed that the developed system contained various cell types such as smooth muscle cells and ECs, as well as CMs, but not CFs. Although complete cell composition was not achieved in this study, the authors demonstrated that the combination of 3D bioprinting and organoid technology has the potential to advance the level of complexity of *in vitro* tissue models.

The organs in our body do not independent but interact continuously constantly interact with other organs and the environment through a variety of signals such as electricity, biomolecules, and endocrine signals. In this regard, many efforts have been made to develop multi-organ systems (organs-on-a-chip), and future breakthroughs in 3D bioprinting technology would contribute to the advancement of these initiatives. To achieve this, bioprinted tissue research should contemplate the inclusion of various channels (e.g., nerves, blood vessels, lymph vessels) to enable the interconnection of tissue models. This approach may facilitate the study of multi-organ crosstalk including absorption, distribution, metabolism, and elimination of drugs or biomolecules, as well as cancer metastasis.

Assessment techniques for *in vitro* tissue models play a key role in translating developed tissue models into clinical applications. Therefore, the assessment techniques must also be advanced to validate volumetric and large-scale tissue models according to the development of 3D tissue models (Yong et al., 2020). For example, echocardiography (a clinical procedure to verify cardiac function) has been applied to measure volume changes in an *in vitro* cardiac chamber model (Li et al., 2018; Macqueen et al., 2018; Kupfer et al., 2020). Other studies have validated bioprinted tissues using light-sheet microscopy and the tissue-clearing method. Importantly, these methods enable the non-destructive visualization and monitoring of volumetric samples and bioprinted constructs (Ding et al., 2018; Yong et al., 2020).

In terms of regulation, it is inadequate to determine the predictive capacity by comparing the gold standards used for *in vivo* testing with the results from *in vitro* tissue models on a one-to-one basis (Pridgeon et al., 2018). Thus, it may be reasonable to establish a new gold standard by integrating various predictive approaches including *in silico* models. In fact, the ongoing comprehensive *in vitro* proarrhythmia assay (CiPA) project aims to develop a new *in vitro* paradigm that can more accurately predict the cardiotoxicity of new drugs (Strauss et al., 2021). Therefore, these advanced *in vitro* tissue models must be extensively validated and documented to enable their widespread adoption in clinical applications.

## Author Contributions

DGH and YC wrote the original draft and prepared the figures. JJ supervised the work and revised the draft. All authors contributed to manuscript revision, read, and approved the submitted version.

## Conflict of Interest

The authors declare that the research was conducted in the absence of any commercial or financial relationships that could be construed as a potential conflict of interest.
