# Supplementary material for: 3D Bioprinting-Based Vascularized Tissue Models Mimicking Tissue-Specific Architecture and Pathophysiology for in vitro Studies
Source: Front Bioeng Biotechnol. 2021 May 31;9:685507. doi: 10.3389/fbioe.2021.685507 (PMC8201787; doi:10.3389/fbioe.2021.685507)
Supplement: Supplementary file 1 [file Table_1.docx]

**Supplementary table 1.** Structural features of 3D bioprinted in vitro tissue models

| **Tissue** | **Structural feature** | **Cell sources** | | **Printing approach** | **Ref.** |
| --- | --- | --- | --- | --- | --- |
|  |  | **Parenchymal cell** | **Stromal cell** |  |  |
| Heart | Patch-like structure | NRCM, hiPSC-CM | HUVEC | Extrusion-based printing, seeding CMs into 3D bioprinted endothelialized scaffold | (Zhang et al., 2016) |
|  |  | hiPSC-CM | HUVEC | Microfluidic-based printing | (Maiullari et al., 2018) |
|  | Anisotropic alignment of cardiomyocytes | NRCM | - | Extrusion-based printing | (Wang et al., 2018) |
|  |  | hiPSC-CM, AC16 | HCF | Extrusion-based printing, visible light cross-linker | (Anil Kumar et al., 2019) |
|  |  | NRCM | - | Extrusion-based 3D bioprinting | (Das et al., 2019) |
|  |  | hiPSC-CM | - | DLP-based printing | (Yu et al., 2019) |
|  | Ring/cylindrical structure | hiPSC-CM | HUVEC, NHDF | Kenzan method | (Arai et al., 2018) |
|  |  | hiPSC-CM | hMSC, HCF | Deposit cardiac spheroids within self-healing hydrogel bath | (Daly et al., 2021) |
|  | Chamber-like structure | C2C12, hES-CM | HUVEC | Extrusion-based printing, supporting bath | (Lee et al., 2019a) |
|  |  | NRCM, hiPSC-CM | HUVEC | Extrusion-based printing, supporting bath | (Noor et al., 2019) |
|  |  | hiPSC-CM | - | Extrusion-based printing, supporting bath | (Kupfer et al., 2020) |
| Liver | Spheroid | HepG2/C3A | - | Extrusion-based printing, spheroid printing | (Bhise et al., 2016) |
|  | Planar/cubical structure | HPC | HSC, HUVEC | Extrusion-based printing | (Norona et al., 2016) |
|  |  | HPC | HSC, HUVEC | Extrusion-based printing | (Nguyen et al., 2016) |
|  |  | HPC | HSC, HUVEC, Kupffer | Extrusion-based printing | (Norona et al., 2019) |
|  |  | HPC | Mouse fibroblast | Kenzan method | (Kizawa et al., 2017) |
|  |  | HPC | Hematopoietic stem cells | Kenzan method | (Ide et al., 2020) |
|  | Liver sinusoid | HepaRG | HUVEC | Extrusion-based printing | (Lee et al., 2019b) |
|  |  | HepG2/C3A | HUVEC | Extrusion-based printing, fugitive biomaterial ink for vascular channel | (Massa et al., 2017) |
|  |  | HepaRG | HUVEC, LX2 | Extrusion-based printing | (Lee et al., 2020) |
|  | Hepatic lobule structure | hiPSC-HPC | HUVEC, ADSC | DLP-based printing | (Ma et al., 2016) |
|  |  | HepaRG | SteCs | DLP-based printing | (Grix et al., 2018) |
|  |  | hiPSC-HPC | - | DLP-based printing | (Yu et al., 2019) |
|  |  | HepG2/C3A, | EA.hy 926 | Extrusion-based printing, preset cartridge | (Kang et al., 2020) |
|  |  | hiHep | - | DLP-based printing | (Mao et al., 2020) |
| Kidney | Proximal tubule | RPTEC/TERT1 | HNDF | Extrusion-based printing, fugitive biomaterial ink | (Homan et al., 2016) |
|  | Proximal tubule with vascular interface | RPTEC/TERT1, cAP-0004 | - | Extrusion-based printing, fugitive biomaterial ink | (Lin et al., 2019) |
|  |  | HK-2, PCS-400-010 | hBMMSC, HUVEC | Extrusion-based printing, co-axial cell printing | (Singh et al., 2020) |
| Blood vessel | Linear structure | HUVEC | - | Solid freeform fabrication | (Lee et al., 2014a) |
|  |  | HUVEC | HNLF | Extrusion-based printing, layer-by-layer approach | (Lee et al., 2014b) |
|  |  | EPC | - | Extrusion-based printing, co-axial cell printing | (Gao et al., 2017) |
|  | Multi-channel | HUVEC | MC3T3 | Extrusion-based printing | (Bertassoni et al., 2014) |
|  |  | HUVEC | 10T1/2 MF, HNDF | Extrusion-based printing | (Kolesky et al., 2014) |
|  |  | HUVEC | hMSC, HNDF | Extrusion-based printing | (Kolesky et al., 2016) |
|  |  | - | - | Stereolithography | (Grigoryan et al., 2019) |
|  | Convoluted structure | HUVEC | hAEC, HL-60 | Extrusion-based printing  Co-axial cell printing | (Gao et al., 2018) |
|  | Multi-layered structure | HUVEC, HAoSMC | - | Extrusion-based printing  Triple-coaxial cell printing | (Gao et al., 2019) |
|  |  | HUVEC, HCASMC | HDF, THP-1 | Extrusion-based printing, In-bath co-axial cell printing | (Gao et al., 2020) |
|  | Capillary-sized microvessel | HUVEC | Human bone marrow‐derived hS5 stromal cell | Multiphoton lithography, photodegradable material | (Arakawa et al., 2017) |
| Intestine | Intestinal barrier | Caco-2 | hIMF | Extrusion-based printing, print on transwell | (Madden et al., 2018) |
|  | Intestinal villi | Caco-2 | - | Extrusion-based printing | (Kim and Kim, 2018) |
|  |  | Caco-2 | HUVEC | Extrusion-based printing | (Kim and Kim, 2020) |
| Lung | Alveolar barrier | A549 | EA.hy 926, HUVEC | Valve-based printing, print on transwell | (Horvath et al., 2015) |
|  |  | NCI-H1703, NCI-H441 | MRC5, HULEC-5a | Inkjet-based printing, print on transwell | (Kang et al., 2021) |
|  | Airway epithelium | Human tracheal epithelial cell | hDMEC, hLF | Extrusion-based printing | (Park et al., 2019) |
| Skin | Dermis | Primary human Skin cell | NIH 3T3 | Extrusion-based printing | (Pourchet et al., 2017) |
|  | Epidermis + Dermis | FB, KC | - | Valve-based printing | (Lee et al., 2014c) |
|  |  | HDF, HEK | - | Extrusion-based printing (HDF), inkjet-based printing (HEK), Print on transwell | (Kim et al., 2018) |
|  |  | Primary KC, primary FB | HECFC-derived EC, Primary PC | Valve-based printing | (Baltazar et al., 2020) |
|  | Epidermis + dermis + hypodermis + Vascular channel | HDF, HEK, HPA | HUVEC | Extrusion-based printing (HDF, HPA), inkjet-based printing (HEK), fugitive biomaterial ink for vascular channel | (Kim et al., 2019) |
|  |  | nHDF, dHDF, nHEK,  nHPA, dHPA | HUVEC | Extrusion-based printing (nHDF,dHDF, nHPA, dHPA),  inkjet-based printing (nHEK),  Co-axial cell printing (HUVEC) | (Kim et al., 2021) |
